# White noise enhances new-word learning in healthy adults

**DOI:** 10.1038/s41598-017-13383-3

**Published:** 2017-10-12

**Authors:** Anthony J. Angwin, Wayne J. Wilson, Wendy L. Arnott, Annabelle Signorini, Robert J. Barry, David A. Copland

**Affiliations:** 1 University of Queensland, School of Health and Rehabilitation Sciences, Brisbane, Australia; 2 Hear and Say, Brisbane, Australia; 3 University of Wollongong, School of Psychology and Brain & Behaviour Research Institute, Wollongong, Australia; 4 University of Queensland, UQ Centre for Clinical Research, Brisbane, Australia

## Abstract

Research suggests that listening to white noise may improve some aspects of cognitive performance in individuals with lower attention. This study investigated the impact of white noise on new word learning in healthy young adults, and whether this effect was mediated by executive attention skills. Eighty participants completed a single training session to learn the names of twenty novel objects. The session comprised 5 learning phases, each followed by a recall test. A final recognition test was also administered. Half the participants listened to white noise during the learning phases, and half completed the learning in silence. The noise group demonstrated superior recall accuracy over time, which was not impacted by participant attentional capacity. Recognition accuracy was near ceiling for both groups. These findings suggest that white noise has the capacity to enhance lexical acquisition.

## Introduction

Stochastic resonance (SR), a phenomenon whereby signal processing is enhanced by the addition of random noise, has been widely demonstrated across various modalities, including visual, auditory, tactile and cross-modal forms of processing^1,2^. Of particular interest are findings that auditory noise has the capacity to enhance some aspects of human cognitive performance, such as the speed of arithmetical computations^3^. Research also suggests that the effects of noise may be mediated by the attentional capacity of participants. For instance, in children with attention deficit hyperactivity disorder (ADHD), listening to white noise has been shown to improve performance on memory^4^ and go/no-go tasks^5^, as well as improve speech recognition thresholds^6^. White noise has also been shown to improve cognitive performance in typically developing school children rated as inattentive by their teachers, and worsen performance in those rated as attentive or highly attentive^7,8^.

Such findings may be consistent with the moderate brain arousal (MBA) model^9^, which postulates a link between dopamine function and the effects of white noise on cognitive performance. The model proposes that suboptimal dopamine levels, such as may be found in people with ADHD, can result in reduced levels of neural noise which may impact cognitive performance. In such cases, the provision of a moderate level of white noise can optimize cognitive performance by increasing neural noise via the perceptual system. The model therefore predicts that adding white noise can be beneficial to cognitive performance in those with lower attention, but that the same level of noise may impair performance in those with normal attention where neural noise levels are already optimal.

Recent neuroimaging evidence supports the notion that dopamine may underpin the modulatory influence of white noise on cognitive performance. Rausch *et al*.^10^ demonstrated that the provision of white noise during the encoding of scene images resulted in mild improvements to subsequent recognition memory. More importantly, their analysis of functional magnetic resonance imaging (fMRI) data revealed that white noise decreased sustained activity and increased event-related activity within the substantia nigra and ventral tegmental area, and increased functional connectivity between those regions and the superior temporal sulcus. Based on these findings, Rausch *et al*. suggested that white noise enhances phasic dopamine release, thereby modulating activity within the superior temporal sulcus and leading to increased attention and memory formation.

It should be noted, however, that white noise does not benefit all aspects of cognitive performance. In a series of experiments examining the effects of white noise on dopamine dependent cognitive functions in healthy adults, Herweg and Bunzeck^11^ found that white noise selectively impaired working memory when presented during the maintenance phase of the task relative to performance in silence or when listening to a pure tone signal. White noise had no impact on working memory accuracy when played during both the encoding and maintenance phases or continually throughout the task. In contrast, in a long term memory task, white noise was observed to facilitate the speed of perceptual judgements during encoding, but had no impact on subsequent recognition memory performance.

Taken together, although white noise does not provide a general enhancement for all aspects of cognition, there is evidence to suggest that it can improve some aspects of cognitive performance via modulation of dopaminergic circuitry. If this notion is correct, then the effects of white noise on cognitive performance may be expected to mirror those observed from the effects of dopamine itself. The administration of levodopa, an exogenous dopamine precursor, has been shown to modulate performance on various cognitive tests. The nature of its effects varies according to the nature of the task, with observations including impairments to reversal learning^12^, improvements to feedback-based procedural learning^13^ and alterations to semantic processing^14,15^. There is also evidence that dopamine can improve word learning. For instance, new word learning for existing (familiar) objects in healthy adults may be boosted by dexamphetamine^16,17^ or levodopa^18,19^. More recently, Shellshear *et al*.^20^ examined the impact of levodopa on novel word learning for unfamiliar objects, observing improved recall for participants on levodopa relative to placebo.

In light of these findings, the primary aim of the present study was to identify whether white noise facilitates new word learning in healthy adults. A secondary aim was to explore whether the effect of white noise on learning is mediated by attentional capacity. In particular, the executive control of attention, which relates to conflict resolution processes^21^, was of interest. Kapa *et al*.^22^ recently found that the executive network (conflict effect) score from the Attention Network Test^21^ (ANT) was a significant predictor of artificial language learning in healthy adults, such that those with better executive network scores showed better learning. Accordingly, the present study utilized the ANT to determine if executive control of attention mediates the impact of white noise on novel word learning in healthy adults.

A tertiary aim of the present study was to determine whether the presence of semantic information during learning modulates the impact of white noise. Shellshear *et al*.^20^ found that levodopa was associated with improved recognition accuracy at a one month follow-up for items that had been learned with a semantic description, but not for items learned without a description. These findings appear consistent with dopamine’s capacity to focus semantic activation, as demonstrated by semantic priming studies in healthy adults^15,23^. However, Shellshear *et al*. also found that for both the levodopa and placebo groups, recall accuracy during learning was worse for items learned with a semantic description relative to no description. These findings mirrored those of Whiting *et al*.^24^, who also found poorer learning for items learned with a semantic description, leading them to suggest that the presence of too much semantic information may be counterproductive to learning when participants are under limited time constraints. Addressing such issues, Angwin *et al*.^25^ recently demonstrated that pairing novel words with just two semantic attributes (e.g., Sticky Loud), rather than a lengthy description, resulted in superior recognition during learning relative to items that had been paired with meaningless names of a similar length (e.g., Mansey Smeath). On that basis, the present study used a learning paradigm similar to Angwin *et al*. in order to determine whether white noise would better facilitate the learning of items paired with semantic information relative to items paired with unfamiliar names.

In summary, the present study sought to determine whether white noise facilitates new word learning, whether the effects of white noise are mediated by the executive control of attention, and whether any effect of white noise is more prominent for words linked to semantic information. The following hypotheses were proposed. Firstly, consistent with reports that the beneficial effects of white noise may be mediated by dopaminergic circuitry^10^, together with the beneficial effect of dopamine on word learning^20^, it was predicted that word learning would be improved in white noise relative to silence. Secondly, it was predicted that any improvements to word learning in the presence of white noise would be related to attention, being larger in persons with lower executive control of attention. Thirdly, given that dopamine is known to facilitate semantic processing^15^, it was predicted that white noise would facilitate learning of items paired with semantic information more than items paired with no semantic information.

## Methods

### Participants

Ninety-six undergraduate students participated in the study for course credit. Fourteen participants were ineligible for study inclusion, as 7 reported English as their second language, 1 indicated impaired vision, 2 failed a hearing screen, and 4 were taking anti-depressant medication. The 82 remaining participants consisted of only two males, and so these two male participants were also excluded in order to obtain a more homogeneous sample. The 80 eligible participants were assigned to complete the experimental task either in noise (n = 40) (Age *M* 21.02, *SD* 4.46 years; Education *M* 14.6, *SD* 4.46 years) or in silence (n = 40) (Age *M* 21.35, *SD* 4.19 years; Education *M* 15.05, *SD* 1.31 years). Age and education were not significantly different between groups. All eligible participants reported English as their first language and normal or corrected to normal vision. All participants also reported as right handed, with the exception of 2 left handed participants in the silence group and 1 in the noise group. A hearing screen indicated that all eligible participants presented with normal hearing sensitivity (thresholds equal to or less than 20db) in both ears across 500, 1000, 2000 and 4000 Hz. The project was approved by The University of Queensland Medical Research Ethics committee and was performed in accordance with the relevant guidelines and regulations. Participants provided written informed consent prior to the commencement of testing.

#### Learning Task

Stimuli consisted of a subset of the items utilized in Angwin *et al*.^25^. Specifically, coloured pictures of 20 unique aliens developed by Gupta *et al*.^26^ for word learning studies were used. Each alien was then paired with a unique, three word name, with 10 aliens assigned to a semantic (SEM) condition and 10 assigned to a name (NAM) condition. In both the SEM and NAM conditions, the first name was always a legally spelled nonword (e.g., Blags). These nonwords were generated from the ARC Nonword database (4–6 letters in length, neighbourhood size between 1 and 3, and summed frequency of neighbours between 7 and 60)^27^. In the SEM condition, the nonword name was combined with two adjectives (e.g., Blags Awful Fickle). These adjective pairs were formed from 20 adjectives (4–6 letters in length) selected from the Medical Research Council (MRC) Psycholinguistic Database^28^, which were randomly combined to form 10 pairs. In any instances where a pair contained inconsistent adjectives (e.g., Loud Quiet), the items were re-paired with other adjectives. In the NAM condition, the nonword name was combined with two proper names (e.g., Zalph Jerram Tofts). The proper names were twenty uncommon English surnames selected from the British Census for the years 1538 to 2005. As reported in Angwin *et al*.^25^, the surnames chosen were judged as uncommon in Australia by three Bachelor of Speech Pathology graduates and these names were then randomly paired to produce 10 proper name pairs.

### Procedure

Participants completed a single computerized training session of approximately one hour duration. The training session consisted of a series of 5 learning phases, each followed immediately by a recall task. After the last recall task had been completed, participants then completed a final recognition task. All components were presented using E-Prime 2.0 software (Psychology Software Tools, Pittsburgh, PA).

During each of the five learning phases, participants were presented with the 20 alien/name pairs, one at a time on the computer screen. Each alien/name pair was presented twice, such that participants had viewed each pair a total of 10 times by the end of the training session. The order of stimulus presentation was randomized separately for each learning phase and participant. Trials consisted of a fixation point ‘+’ presented in the middle of the screen for 500 ms, followed by an alien picture with its three word name written underneath for 10 seconds.

Following each learning phase, participants completed a recall task. During the recall task, the 20 alien pictures were presented one at a time in a random order in the middle of the computer screen. For each picture, participants were instructed to use the computer keyboard to type the first name of the alien (i.e., the nonword name) and then press the ‘enter’ key. Each picture remained on screen until the enter key was pressed, and then the next alien was automatically displayed.

After the 5^th^ recall task the participants completed a final recognition task. Pictures of the same 20 aliens from the learning phases were displayed one at a time together with either their correct three word name or the full three word name of another alien viewed in the session. Participants used the computer mouse to indicate whether the names were correct or not by pressing the left button for correct alien/name pairs and the right button for incorrect pairs. After each response, the next trial was displayed automatically.

For participants in the noise group, white noise was presented via AKG K550 closed back reference class headphones (circumaural, frequency response 12 Hz to 28 kHz, impedance 32 ohms, sensitivity 114 dB SPL/V) at 70 dB SPL(A) during each of the five learning phases. This output level was set as the level produced when playing the white noise through the headphones placed on an artificial ear coupled to a sound level meter. The artificial ear was a Brüel & Kjær (B&K) type 4153 with a B&K DB 0843 adaptor and a B&K type 4144 1″ pressure-field microphone. The sound level meter coupled to the microphone was a B&K type 2250 Hand-held Analyzer (class 1) set to record on a slow (1 s) setting. The white noise (mono track, 44100 Hz sampling frequency with 32-bit float) played through the headphones was generated using Audacity software (version 2.0.5 for windows generating white noise with a bandwidth from 86 Hz to 22.007 kHz) running on Dell Optiplex 9020 desktop computers (Intel Core i5–2400 CPU @ 3.1 GHz with 4 GB RAM) running Windows 7 (SP1, 64 bit) with Realtek High Definition Audio drivers (version 6.0.1.5883). Participants removed the headphones when performing the recall and recognition tasks. Participants in the silence group were not required to wear headphones.

### Attention testing

On a day separate from the learning session, participants completed a modified version of the Attention Network Test (ANT)^21^ to assess their executive control of attention. Trials consisted of a central arrow, pointing left or right, surrounded by flanking arrows in either the same (congruent) or the opposite (incongruent) direction. Participants were required to identify the direction of the central arrow as quickly and accurately as possible by pressing the left or right arrow key on the computer keyboard. Target stimuli were preceded by different cues on some trials. By comparing reaction times for incongruent compared to congruent trials, the extent to which the incongruent flanking arrows interfere with task performance can be determined. Accordingly, the conflict (executive network) effect score is calculated by subtracting the mean reaction time for congruent trials from the mean reaction time for incongruent trials (only trials with a correct response are included in this calculation). This measure provides an indication of the efficiency of the executive control network, such that the executive control of attention is lower in those people with a larger conflict effect score.

Stimuli were presented across three blocks of 48 trials, with the order of stimuli randomized within each block. Short rest breaks were provided to participants after each block. Prior to commencing the test, participants completed a practice task consisting of 12 trials. Feedback on reaction time and accuracy was provided during this practice task, however no feedback was provided during the test proper. The task was presented using E-prime 2.0.

### Data analysis

Since the recall and recognition accuracy data were proportional, an arcsine square root transformation was applied to improve variance^29^. For the recall data, skewness was subsequently between −2 and 2 and kurtosis between −2.5 and 2.5 across all recall trials for each condition and group. A repeated measures analysis of variance (ANOVA) was used to analyse the recall accuracy data, with noise (noise, silence) as a between subjects factor, and condition (SEM, NAM) and trial (phase 1–5) as within subjects factors. The conflict effect score was subsequently added to this ANOVA as a covariate, in order to confirm whether any effects of noise were mediated by attention. Four participants (silence n = 1; noise n = 3) were excluded from this covariate analysis due to an exceptionally high error rate on the incongruent condition of the ANT, which precluded a reliable conflict effect for those participants and raised doubts as to whether they had understood the task instructions correctly. A t-test confirmed that the mean conflict effect score for the silence group (*M* 99 ms; *SD* 39) did not differ to the score for the noise group (*M* 96 ms; *SD* 41) (p = 0.728). Although uncorrected degrees of freedom are reported for the ANOVA, whenever Mauchly’s test of sphericity was violated, the Huynh-Feldt adjustment was applied. For the recognition accuracy data, since skewness and kurtosis were both more than ±2.5, Mann Whitney U tests were used to analyse whether noise influenced recognition accuracy for either the SEM or NAM condition.

## Results

### Recall data

**Figure 1:**
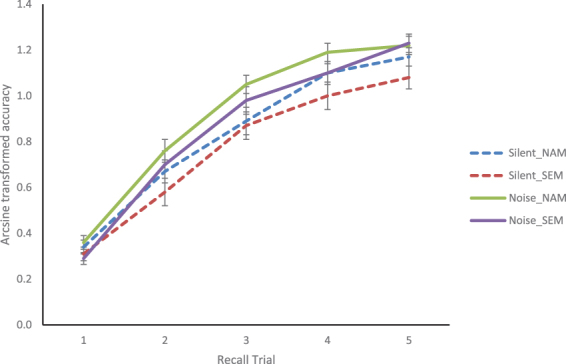
Arcsine transformed proportion accuracy for each group and condition. Standard error bars provided.

Analysis of the recall data revealed a main effect of trial *F*(4,312) = 487.71, *p* < 0.001, η_p_^2^ = 0.862, indicating increased recall accuracy across the learning sessions (Fig. 1). A main effect of condition *F*(1,78) = 24.18, *p* < 0.001, η_p_^2^ = 0.237 was also evident, indicative of better overall recall for the NAM condition relative to the SEM condition. Although there was no main effect of noise, there was a significant noise X trial interaction *F*(4,312) = 2.80, *p* = 0.035, η_p_^2^ = 0.035. This interaction was driven by a difference in the slope of the learning curve between the two groups, as evidenced by a significant quadratic effect for the noise X trial interaction (*F*(1,78) = 4.45, *p* = 0.038, η_p_^2^ = 0.054; the linear effect was not significant *p* = 0.089), demonstrating better learning over time for the noise group relative to the silence group. No other interactions were significant, although the 3 way interaction between noise, trial and condition approached significance (*p* = 0.073).

In order to determine whether the noise x trial interaction was modulated by executive attention, the ANT conflict effect score was added as a covariate to the ANOVA. This analysis revealed no significant effect of the attention covariate (p = 0.135), and confirmed that the noise X trial interaction was still significant *F*(4,292) = 2.67, *p* = 0.041, η_p_^2^ = 0.035 and still driven by a difference in the slope of the learning curves (quadratic effect *F*(1,73) = 4.88, *p* = 0.030, η_p_^2^ = 0.063; the linear effect was not significant *p* = 0.209).

### Recognition accuracy

Analysis of the recognition accuracy data with Mann Whitney U tests revealed no significant effect of noise on performance for either condition. Mean accuracy was over 90% for both groups across the SEM and NAM conditions (Silence group: SEM condition *Mean* = 1.32, *SD* = 0.21, NAM condition *M* = 1.31, *SD* = 0.19; Noise group: SEM condition *M* = 1.28, *SD* = 0.26, NAM condition *M* = 1.30, *SD* = 0.29).

## Discussion

This study aimed to examine the impact of white noise on new word learning in healthy adults and determine whether any observed effects are mediated by attention. It was hypothesized that white noise would improve word learning, and that this effect would be most evident for participants with lower executive attention. It was further hypothesized that white noise would have the largest impact on words learned with semantic attributes.

The presence of white noise resulted in superior word learning relative to silence, as evidenced by more effective recall over time for the noise group. Previous research has illustrated that white noise has the capacity to improve recognition memory^10^, the speed of arithmetic calculations^3^ and the speed of perceptual judgements of scene images^11^ in healthy adults (but see also below re null findings). The current results extend these findings, demonstrating that white noise can also boost the lexical acquisition of novel word forms in healthy adults. The findings also provide further evidence for cross-modal stochastic resonance, whereby auditory noise improved cognitive performance on a visual task.

There are a number of neural mechanisms that may underpin the observed effects. Levodopa improves word learning in healthy adults^18,20^, suggesting that dopaminergic mechanisms may be a candidate mechanism. Indeed, recent evidence has shown that successful new word learning is associated with ventral striatal activity^30^, and that dopaminergic activity may be critical to the process as evidenced by increased functional connectivity within the substantia nigra/VTA, ventral striatum and hippocampus^31^. Rausch *et al*.^10^ postulated that white noise improves encoding and memory formation by increasing phasic dopamine release, thereby increasing the salience of externally presented stimuli. Accordingly, we speculate that the improved word learning induced by white noise in the present study may be driven by increased phasic dopamine activity, which facilitates attention and enhances the salience of the alien/word pairs. In addition to facilitating attention, white noise may improve word learning via its impact on working memory, as phasic dopamine activity has been linked to the updating of working memory^32^.

Word learning success in healthy adults is also dependent upon synchronization of left hippocampal activity and ipsilateral association regions^33^. The hippocampus receives a number of dopaminergic inputs including from the VTA in response to novelty detection and reward prediction, contributing to hippocampal-dependent memories^34^. Thus, consistent with findings that white noise modulates activity within the VTA^10^, benefits to word learning may be due to facilitation of hippocampal-dependent memory formation. It is acknowledged that these proposed neural mechanisms are speculative at this point, and further neuroimaging research will be required to verify such claims.

The hypothesis that noise would facilitate learning more effectively in those with lower executive attention was not supported. Specifically, the analysis showed that the improvements to learning in white noise were not modulated by participants’ executive control of attention, as measured by the ANT. This result contrasts with findings that white noise improves performance on memory tasks in children with ADHD^4^ and children rated as sub-attentive^8^, and appears inconsistent with the predictions of the MBA model^9^. However, given that all participants in the present study were healthy undergraduate university students, one potential explanation for this finding is that the range of attentional capacity in this participant cohort was not wide enough to capture an effect of attention.

Research indicates that the optimal dopamine level for task performance may vary across tasks, and that the impact of dopamine manipulation will vary depending on parameters such as the type of task, the brain regions targeted and baseline dopamine levels within those regions^35^. Similarly, the effects of white noise on cognitive function may be expected to vary depending on a range of factors. Herweg and Bunzeck^11^ found that white noise impaired working memory performance in healthy adults if it was selectively presented during the maintenance phase of the task. Moreover, although white noise was found to speed perceptual judgments during the encoding phase of a reward modulated long term memory task, subsequent recognition memory was not affected. Their findings prompt the need for ongoing research to determine which variables contribute to the effects of white noise on word learning as well as other cognitive tasks.

There are two final points worthy of consideration. Firstly, despite inducing improvements to recall accuracy, white noise had no impact on recognition accuracy. This finding is understandable given that recognition accuracy was already near ceiling for the silence group. Research suggests that recognition is typically less difficult than recall^36^, and recognition was only assessed after the final recall phase in the present study. A more frequent assessment of recognition accuracy throughout learning is warranted in future studies in order to further explore the impact of white noise on recognition processes. Secondly, regardless of noise, recall accuracy for items learned in the semantic condition was significantly worse than items learned without semantic information, whilst recognition accuracy was similar for both conditions. Although previous studies of word learning have also found worse recall for items paired with semantics^20,24^, their semantic information consisted of more lengthy semantic descriptions. Consistent with the present study, Angwin *et al*.^25^ restricted the semantic information to only two adjectives and observed no impact of semantic information on recall, together with better recognition in the semantic condition at each learning session. Shellshear *et al*.^20^ also observed better recognition of items learned with semantic information at a one month follow-up for participants on levodopa. Accordingly, the negative impact of semantic information on learning in the present study was unexpected, and might indicate that the semantic attributes distracted participants and reduced attention towards the nonword names.

## Conclusions

The presence of white noise significantly enhanced new word learning in healthy adults, but this effect was not influenced by participant executive attention. The effects of white noise are potentially underpinned by modulation of dopaminergic circuitry and subsequent facilitation of attention and memory, however direct measurement of dopamine uptake (e.g., using Positron Emission Tomography) would be required to confirm these suggestions. The findings provide the impetus for additional research into the potential use of white noise as a non-pharmacological treatment for people with dopaminergic dysregulation or loss, and for people with language or learning difficulties.

## References

[1] McDonnell MD, Ward LM (2011). The benefits of noise in neural systems: bridging theory and experiment. Nat. Rev. Neurosci..

[2] Moss F, Ward LM, Sannita WG (2004). Stochastic resonance and sensory information processing: a tutorial and review of application. Clin. Neurophysiol..

[3] Usher M, Feingold M (2000). Stochastic resonance in the speed of memory retrieval. Biol. Cybern..

[4] Soderlund G, Sikstrom S, Smart A (2007). Listen to the noise: noise is beneficial for cognitive performance in ADHD. J. Child Psychol. Psyc..

[5] Baijot S (2016). Neuropsychological and neurophysiological benefits from white noise in children with and without ADHD. Behav. Brain Funct..

[6] Soderlund GBW, Jobs EN (2016). Differences in speech recognition between children with attention deficits and typically developed children disappear when exposed to 65 dB of auditory noise. Front. Psychol..

[7] Helps SK, Bamford S, Sonuga-Barke EJS, Soderlund GBW (2014). Different effects of adding white noise on cognitive performance of sub-, normal and super-attentive school children. Plos One.

[8] Soderlund GBW, Sikstrom S, Loftesnes JM, Sonuga-Barke EJS (2010). The effects of background white noise on memory performance in inattentive school children. Behav. Brain Funct..

[9] Sikstrom S, Soderlund G (2007). Stimulus-dependent dopamine release in attention-deficit/hyperactivity disorder. Psychol. Rev..

[10] Rausch VH, Bauch EM, Bunzeck N (2014). White noise improves learning by modulating activity in dopaminergic midbrain regions and right superior temporal sulcus. J. Cognitive Neurosci..

[11] Herweg NA, Bunzeck N (2015). Differential effects of white noise in cognitive and perceptual tasks. Front. Psychol..

[12] Vo A, Seergobin KN, Morrow SA, MacDonald PA (2016). Levodopa impairs probabilistic reversal learning in healthy young adults. Psychopharmacology.

[13] de Vries MH, Ulte C, Zwitserlood P, Szymanski B, Knecht S (2010). Increasing dopamine levels in the brain improves feedback-based procedural learning in healthy participants: An artificial-grammar-learning experiment. Neuropsychologia.

[14] Angwin AJ (2004). Dopamine and semantic activation: An investigation of masked direct and indirect priming. J. Int. Neuropsych. Soc..

[15] Copland DA, McMahon KL, Silburn PA, de Zubicaray GI (2009). Dopaminergic neuromodulation of semantic processing: A 4-T fMRI study with levodopa. Cereb. Cortex.

[16] Breitenstein C (2004). D-amphetamine boosts language learning independent of its cardiovascular and motor arousing effects. Neuropsychopharmacol..

[17] Whiting E, Chenery HJ, Chalk J, Darnell R, Copland DA (2008). The explicit learning of new names for known objects is improved by dexamphetamine. Brain Lang..

[18] Knecht S (2004). Levodopa: Faster and better word learning in normal humans. Ann. Neurol..

[19] Breitenstein C (2006). A shift of paradigm: From noradrenergic to dopaminergic modulation of learning?. J. Neurol. Sci..

[20] Shellshear L (2015). Levodopa enhances explicit new-word learning in healthy adults: a preliminary study. Hum. Psychopharm. Clin..

[21] Fan J, McCandliss BD, Sommer T, Raz A, Posner MI (2002). Testing the efficiency and independence of attentional networks. J. Cognitive Neurosci..

[22] Kapa LL, Colombo J (2014). Executive function predicts artificial language learning. J. Mem. Lang..

[23] Roesch-Ely D (2006). Dopaminergic modulation of semantic priming in healthy volunteers. Biol. Psychiat..

[24] Whiting E, Chenery H, Chalk J, Darnell R, Copland D (2007). Dexamphetamine enhances explicit new word learning for novel objects. Int. J. Neuropsychoph..

[25] Angwin AJ, Phua B, Copland DA (2014). Using semantics to enhance new word learning: An ERP investigation. Neuropsychologia.

[26] Gupta P (2004). Space aliens and nonwords: Stimuli for investigating the learning of novel word-meaning pairs. Behav. Res. Meth. Ins. C..

[27] Rastle K, Harrington J, Coltheart M (2002). 358,534 nonwords: The ARC NonwordDatabase. Q. J. Exp. Psychol-A..

[28] Wilson M (1988). MRC psycholinguistic database - machine-usable dictionary, version 2.00. Behav. Res. Meth. Ins. C..

[29] Snedecor, G. & Cochran, W. *Statistical Methods*. 8th edn, (Iowa State University Press, 1989).

[30] Ripolles P (2014). The role of reward in word learning and its implications for language acquisition. Curr. Biol..

[31] Ripolles P (2016). Intrinsic monitoring of learning success facilitates memory encoding via the activation of the SN/VTA-Hippocampal loop. Elife.

[32] Hazy TE, Frank MJ, O’Reilly RC (2010). Neural mechanisms of acquired phasic dopamine responses in learning. Neurosci. Biobehav. R..

[33] Breitenstein C (2005). Hippocampus activity differentiates good from poor learners of a novel lexicon. Neuroimage.

[34] McNamara CG, Tejero-Cantero A, Trouche S, Campo-Urriza N, Dupret D (2014). Dopaminergic neurons promote hippocampal reactivation and spatial memory persistence. Nat. Neurosci..

[35] Cools R, D’Esposito M (2011). Inverted-U-shaped dopamine actions on human working memory and cognitive control. Biol. Psychiat..

[36] Haist F, Shimamura AP, Squire LR (1992). On the relationship between recall and recognition memory. J. Exp. Psychol. Learn..

